# Novel Small-Molecule Inhibitors of Hepatitis C Virus Entry Block Viral Spread and Promote Viral Clearance in Cell Culture

**DOI:** 10.1371/journal.pone.0035351

**Published:** 2012-04-24

**Authors:** Glen A. Coburn, Danielle N. Fisch, Sameer M. Moorji, Jean-Marc de Muys, Jose D. Murga, Dorothy Paul, Kathleen P. Provoncha, Yakov Rotshteyn, Amy Q. Han, Dapeng Qian, Paul J. Maddon, William C. Olson

**Affiliations:** Research and Development, Progenics Pharmaceuticals, Inc., Tarrytown, New York, United States of America; Queen's University, Canada

## Abstract

Combinations of direct-acting anti-virals offer the potential to improve the efficacy, tolerability and duration of the current treatment regimen for hepatitis C virus (HCV) infection. Viral entry represents a distinct therapeutic target that has been validated clinically for a number of pathogenic viruses. To discover novel inhibitors of HCV entry, we conducted a high throughput screen of a proprietary small-molecule compound library using HCV pseudoviral particle (HCVpp) technology. We independently discovered and optimized a series of 1,3,5-triazine compounds that are potent, selective and non-cytotoxic inhibitors of HCV entry. Representative compounds fully suppress both cell-free virus and cell-to-cell spread of HCV *in vitro*. We demonstrate, for the first time, that long term treatment of an HCV cell culture with a potent entry inhibitor promotes sustained viral clearance *in vitro*. We have confirmed that a single amino acid variant, V719G, in the transmembrane domain of E2 is sufficient to confer resistance to multiple compounds from the triazine series. Resistance studies were extended by evaluating both the fusogenic properties and growth kinetics of drug-induced and natural amino acid variants in the HCVpp and HCV cell culture assays. Our results indicate that amino acid variations at position 719 incur a significant fitness penalty. Introduction of I719 into a genotype 1b envelope sequence did not affect HCV entry; however, the overall level of HCV replication was reduced compared to the parental genotype 1b/2a HCV strain. Consistent with these findings, I719 represents a significant fraction of the naturally occurring genotype 1b sequences. Importantly, I719, the most relevant natural polymorphism, did not significantly alter the susceptibility of HCV to the triazine compounds. The preclinical properties of these triazine compounds support further investigation of entry inhibitors as a potential novel therapy for HCV infection.

## Introduction

An estimated 180 million people worldwide are infected with hepatitis C virus (HCV) and are at risk of developing severe and potentially life-threatening liver diseases, including chronic hepatitis, cirrhosis and hepatocellular carcinoma. For the past decade, the combination of pegylated interferon-alpha and ribavirin has served as the standard of care (SOC) for the treatment of HCV infection [1], [2]. These therapies exhibit limited efficacy against strains commonly found in the United States and Europe and are associated with significant toxicities [1], [2]. In 2011, two small-molecule inhibitors of the HCV NS3/4a serine protease, Victrelis™/boceprevir (Merck) and Incivek™/telaprevir (Vertex), received regulatory approval for the treatment of HCV infection. In pivotal phase 3 clinical trials, addition of either agent to the combination of pegylated interferon-alpha and ribavirin significantly improved sustained virologic response (SVR) rates and reduced the duration of therapy for many HCV patients [3]–[6]. Despite the recent paradigm shift in HCV therapy, first generation protease inhibitors suffer from significant drawbacks, including the introduction of additional toxicities, inconvenient dosing regimens and documented viral resistance. In addition to these concerns, there continues to be an urgent need to replace pegylated interferon-alpha and ribavirin with novel direct acting anti-virals to drive further improvements in the efficacy, tolerability and duration of HCV therapy.

In light of these considerations, it is crucial that future efforts focus on the development of a wider repertoire of anti-viral agents, including inhibitors with novel mechanisms of action. Viral entry represents an attractive treatment class for HCV and has been validated clinically for a number of human pathogenic viruses. HCV entry is mediated by two viral envelope glycoproteins, E1 and E2, acting in conjunction with five cell surface receptors, CD81 [7], [8], the scavenger receptor class B type 1 (SR-B1) [9], [10], the Niemann-Pick C1-like 1 cholesterol absorption receptor (NPC1L1) [11] and the tight junction proteins claudin-1 [12] and occludin [13]. The mechanism of HCV attachment to the host target cell is poorly characterized and may involve binding to glycosaminoglycans and/or the LDL receptor [14]–[16]. Alternatively, initial binding of virus/lipoprotein complexes to SR-B1 may prime interactions with the other co-receptors [17]. Following virus attachment, HCV particles recruit CD81/claudin-1/occludin complexes through a poorly defined process. Interactions with SR-B1 and CD81 are likely mediated by E2 [10], [18]–[21]. In contrast, direct binding between the viral envelope glycoproteins and the tight junction proteins, claudin-1 and occludin, has not been established [12], [22]. Indeed, recent evidence suggests that the interaction between the HCV envelope and claudin-1 is indirectly mediated by CD81 [23] and may be regulated *via* receptor tyrosine kinase activity [24]. The requirement for sequential interactions between the viral envelope and key host receptors/co-receptors may provide new drug targets that could be exploited by small-molecule inhibitors.

**Figure 1:**
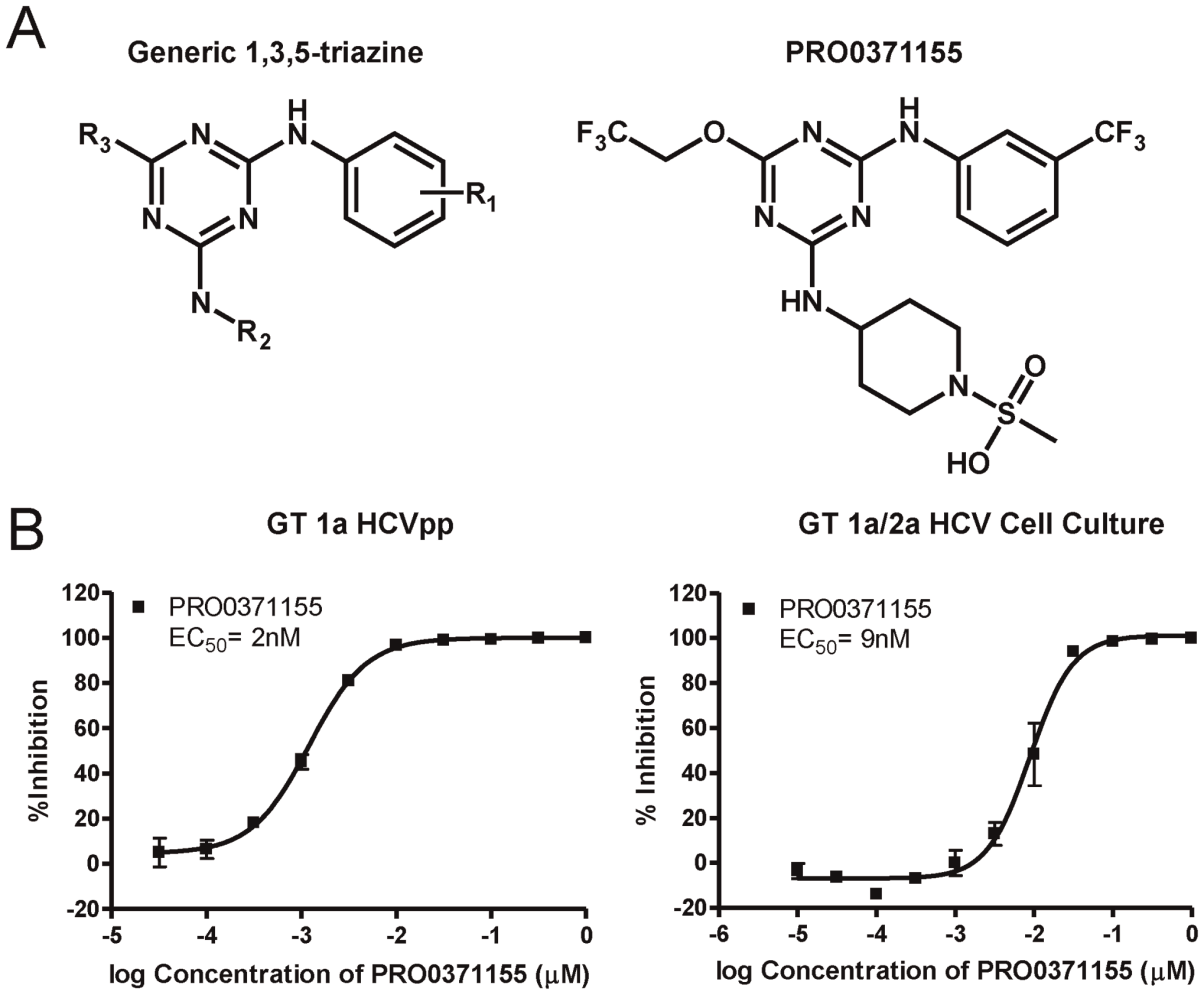
Structure and anti-viral activity of PRO037115. (A, left panel) Generic structure representing the N2-R1-phenyl-N4-R2-6-R3-2,4-diamine-1,3,5-triazine lead series of HCV entry inhibitors. (A, right panel) The structure of PRO0371155. (B, left panel) HCVpp pseudotyped with the GT1a envelope were added to Hep3B cells in the presence of various concentrations of PRO0371155. Luciferase activity was measured 72 hrs post-infection using BrightGlo reagent (Promega) as described in the Materials and Methods. The mean EC_50_ across three independent assays was 2 nM. (B, right panel) PRO0371155 was tested for its inhibitory activity against GT 1a/2a HCV as described in the Materials and Methods. PRO0371155 demonstrated potent anti-viral activity against HCV genotype 1 in this assay: EC_50_ = 9 nM across four independent assays; Positive Control JS-81: EC_50_ = 0.18 µg/mL.

After attachment and co-receptor recruitment, HCV is internalized by receptor-mediated endocytosis *via* clathrin-coated pits into mildly acidic endosomes [25]. The pH-dependence for HCV entry has been well characterized in the HCVpp and HCV cell culture systems with inhibitors that specifically block endosome acidification [7], [26], [27]. By analogy to other closely-related viruses, co-receptor binding and the acidic pH environment of the endosome drive multiple conformational changes that convert the envelope from a metastable state to a lower energy state [28]–[30]. These structural changes result in the exposure of a buried hydrophobic fusion loop which inserts into the host membrane and drives the fusion of the viral envelope with the host membrane, resulting in the delivery of the core particle into the cytoplasm. Compounds that inhibit key intra- or inter-molecular interactions or stabilize intermediate conformations in the HCV envelope may also have the potential to block key HCV fusion processes.

To discover novel small-molecule inhibitors of HCV entry, we optimized and validated an HCVpp-based entry assay for high throughput screening and successfully completed a hit finding campaign of a random library of diversified drug-like compounds. Our screening strategy yielded multiple hit compounds, representing different chemotypes. Chemical optimization of one series led to the discovery of several potent, selective and non-cytotoxic 1,3,5-triazine inhibitors of HCV entry that block both cell-free and cell-cell modes of transmission [31]. Subsequently, a similar series of triazine-based entry inhibitors was reported by a group at Bristol-Myers Squibb [32].

We demonstrate, for the first time, that long-term treatment with an entry inhibitor perturbs normal viral dynamics in culture and leads to complete viral clearance of HCV *in vitro*. We also provide an independent confirmation that a single amino acid variation, V719G, in the transmembrane domain of E2 is sufficient to confer resistance to representative compounds from the triazine series [32]. We have extended these findings by evaluating the fusogenic properties and kinetics of both drug-induced and natural polymorphisms in both the HCVpp and cell culture assays. We show that, isoleucine, the most common natural polymorphism at position 719, does not significantly alter the susceptibility of HCV genotype 1 to triazine compounds. The results indicate that there is a significant fitness cost associated with amino acid variation at position 719 and provide a rationale for the low frequency of pre-existing drug resistant variants in the natural HCV genotype 1 population. Taken together, our data support further investigation of entry inhibitors, including triazines, as a potential therapy for HCV infection.

## Materials and Methods

### HCV pseudoviral particle (HCVpp) entry assay

HCVpp were generated in 293T cells as described previously [7]. Fusogenic envelopes representing different genotypes were isolated and cloned from HCV+ patient sera as described elsewhere [33]. Culture supernatants were harvested 48 h post-transfection and clarified by centrifugation to remove cell debris. The infectivity of the viral supernatants was determined by serial dilution followed by infection of Hep3B cells. The Hep3B (HB-8064) and 293T (CRL-11268) cell lines were obtained from ATCC.

**Table 1:** Various triazine compounds exhibit broad activity against HCV genotype 1a and 1b strains.

Cmpd. ID	HCVpp Median EC_50_ (nM)		HCV Cell Culture EC_50_ (nM)		VSVpp EC_50_ (nM)	Hep3B CC_50_ (nM)
	GT 1a	GT 1b	GT 1a/2a	GT 1b/2a		
PRO0371155	9 (n = 21)	>825 (n = 12)	9	>1,000	39,000	31,000
PRO0373192	9 (n = 18)	10 (n = 17)	11	18	26,357	25,296
PRO0502661	8 (n = 19)	4 (n = 19)	9	12	>100,000	>100,000
PRO0502647	11 (n = 22)	3 (n = 17)	5	3	>100,000	>100,000
PRO0502797	13 (n = 19)	5 (n = 17)	17	11	>100,000	>100,000

HCVpp assays were conducted essentially as described previously [7]. Briefly, Hep3B target cells were plated in 384-well microtiter plates (2,000 cells/well). Test inhibitor compounds were diluted stepwise into the assay medium to the specified final concentration (final DMSO concentration of 0.5%). Diluted virus stock (∼100,000 Relative Light Unit, R.L.U. equivalent) was added and the plates were incubated at 37°C for 72 h. Entry activity was determined using BrightGlo reagent according to the manufacturer's instructions (Promega).

### Isolation and cloning of fusogenic envelopes from HCV+ patient sera

Genotype specific primers and nested RT-PCR were used to amplify the E1/E2 coding sequence from the sera of individuals infected with different HCV 1a, 1b, 2a and 2b strains as described elsewhere [33]. Briefly, viral RNA was isolated from 150 µL of infected patient serum using the QIAamp Viral RNA mini Kit (QIAGEN). Viral RNA was then reverse transcribed using the SuperScript™ III First-Strand Synthesis System for RT-PCR (Invitrogen). The resulting DNA served as template for a first round of amplification with genotype-specific outer primers followed by a second round of amplification with genotype-specific inner primers. The forward inner primer sequence was designed to allow directional cloning of nucleic acids encoding HCV E1/E2 glycoproteins into the pcDNA3.1 TOPO vector (Invitrogen). The forward inner primer also contained a consensus Kozak sequence to direct expression of the envelope glycoproteins. Both rounds of amplification were performed with high fidelity Platinum Pfx DNA polymerase (Invitrogen).

**Figure 2:**
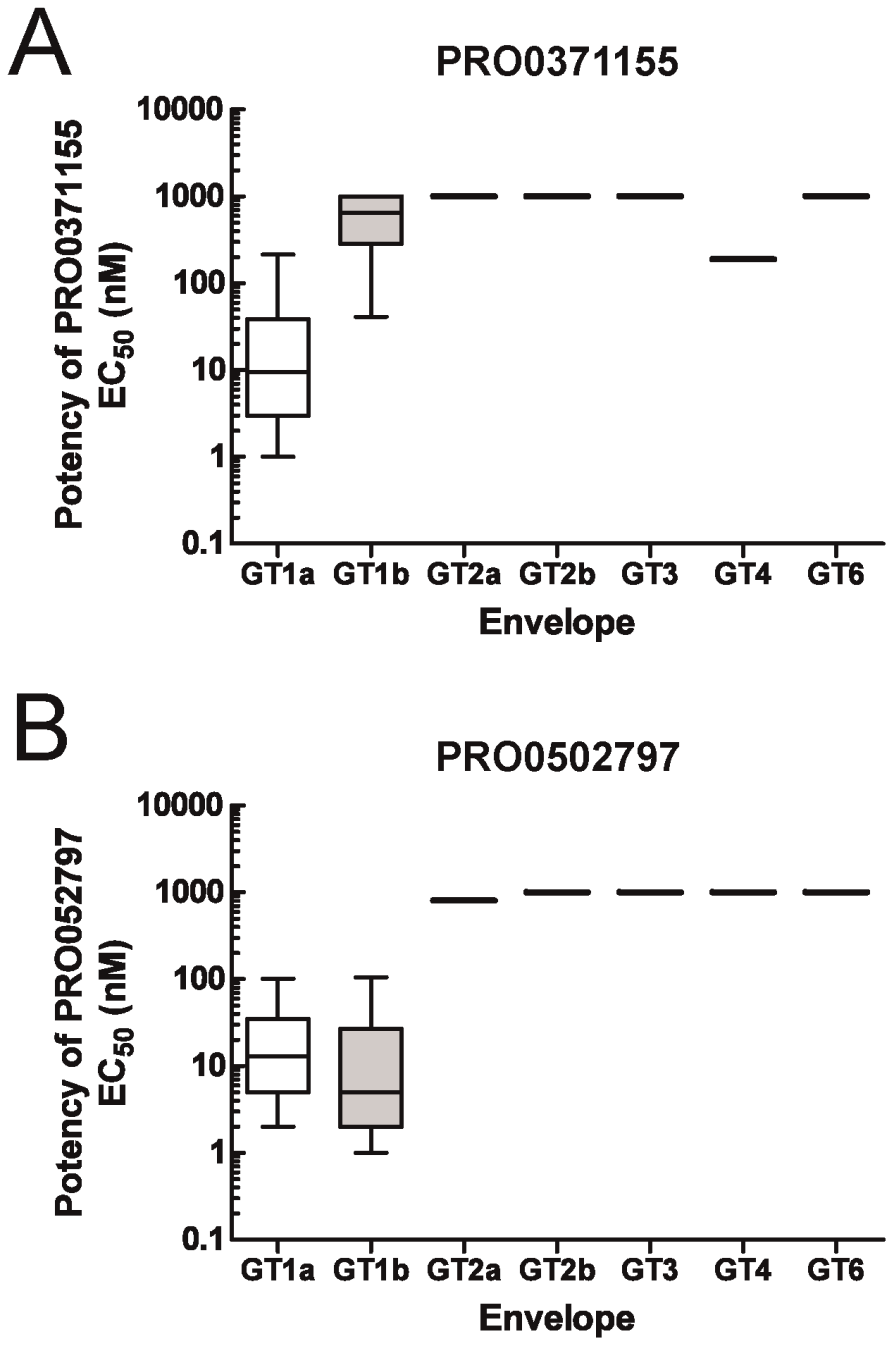
Spectrum of activity of representative triazines against envelopes isolated from HCV^+^ patient sera. HCV RNA was isolated and purified from sera obtained from individuals infected with different strains of HCV, representing genotypes 1a, 1b, 2a, 2b. Envelopes representing genotypes 3, 4, and 6 were described in the Materials and Methods and published elsewhere [33]. Genotype specific primers were used to amplify sequences encoding the E1/E2 glycoprotein from each of the strains by RT-PCR. Envelope sequences were ligated into expression constructs as described in the Materials and Methods. Unique HCVpp, representing different genotypes, were produced in 293T cells and validated with the anti-CD81 mAb, JS-81. Individual HCVpp were normalized based on total infectivity and added to Hep3B cells in the presence of various concentrations of PRO0371155 (Panel A) or PRO0502797 (Panel B) in 384-well microplates. Luciferase activity was measured 72 hrs post-infection using Bright Glo reagent (Promega). The potency of PRO0371155 and PRO0502797 against each fusogenic envelope was expressed as a mean EC_50_. Each data point represents the average of multiple dose response experiments (n>3). The genotype specific panel consisted of the following strains: genotype 1a (n = 22), genotype 1b (n = 19), genotype 2a (n = 2), genotype 2b (n = 1), genotype 3, (n = 1), genotype 4, n = 1), genotype 6 (n = 1). Actual number of strains tested for each compound is summarized in Table 1. The box extends from the 25^th^ to the 75^th^ percentile, with a line at the median EC_50_. The whiskers mark the full range from the lowest to the highest EC_50_.

**Figure 3:**
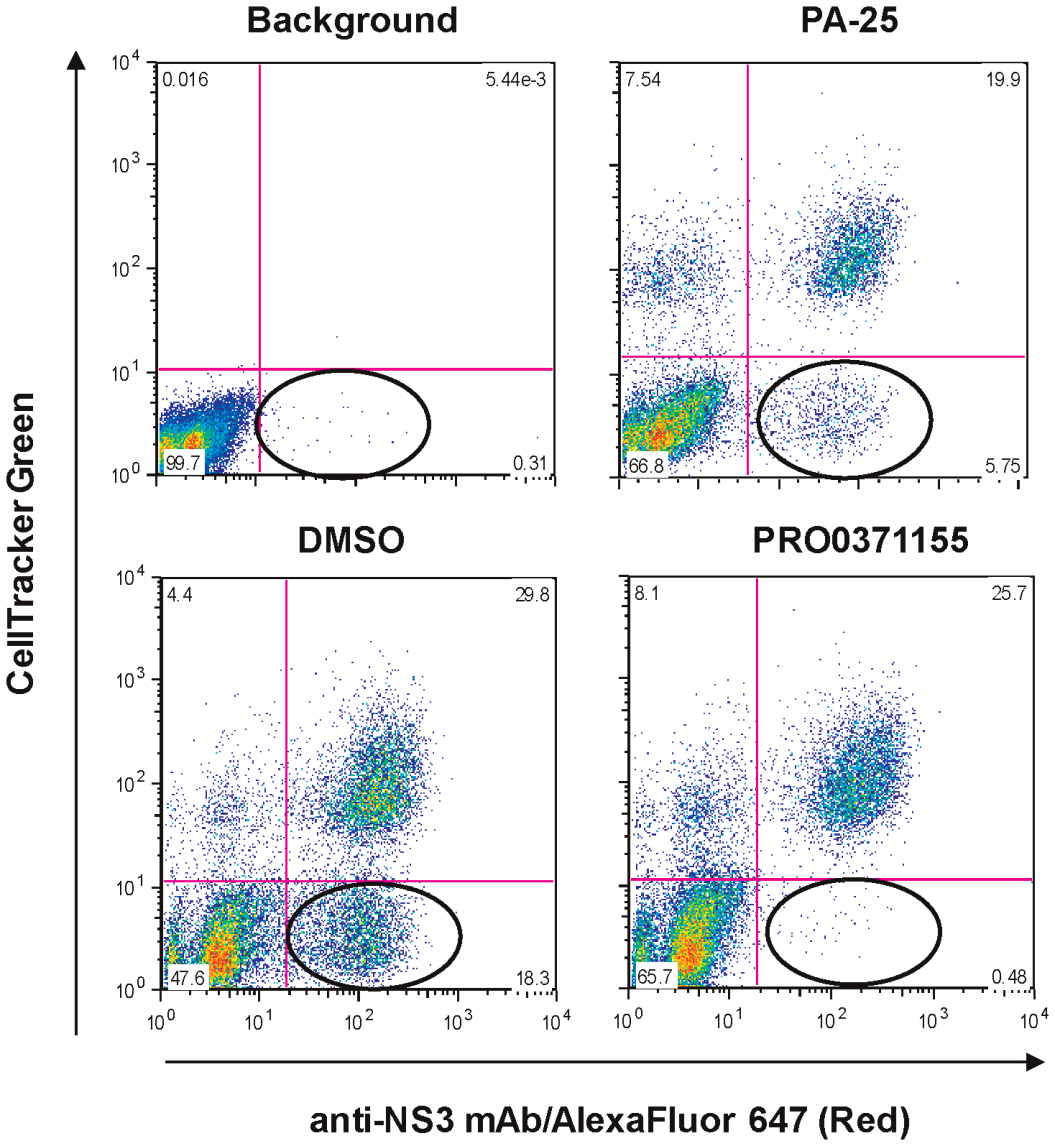
PRO0371155 inhibits HCV infection by both cell-free and cell-cell modes of transmission. The anti-viral activity of PRO0371155 was evaluated in an assay that measures both cell-free and cell-cell transmission of virus [37]. To evaluate transmission of GT 1a/2a HCV in culture, infected cells (90% HCV+) were stained with CMFDA green, according to the manufacturer's instructions (Invitrogen), and mixed at a ratio of 5∶1 with non-stained naïve cells. Mixed infected and naïve cells (7.5×10^5^ total) were seeded in T-75 flasks and subjected to treatment with 1 µM of PRO0371155 or DMSO vehicle for 72 h at 37°C. As a positive control for cell-cell transmission, PA-25, a mouse monoclonal antibody raised against sE2 was also evaluated in the viral spread assay at a single concentration of 10 µg/mL. This concentration is approximately 20-fold above its EC_50_ concentration and is sufficient to completely inhibit HCV entry in a single-cycle infection assay. Cells were detached, fixed and permeabilized with BD Cytofix/cytoperm (BD Biosciences). Infected cells were stained with anti-NS3 antibody 1847 (Virostat) or mIgG1 (BD Biosciences) and counter-stained with 1 µg/mL AlexFluor 647 (Invitrogen). Single- and dual-labeled cells were quantified by flow cytometry.

**Figure 4:**
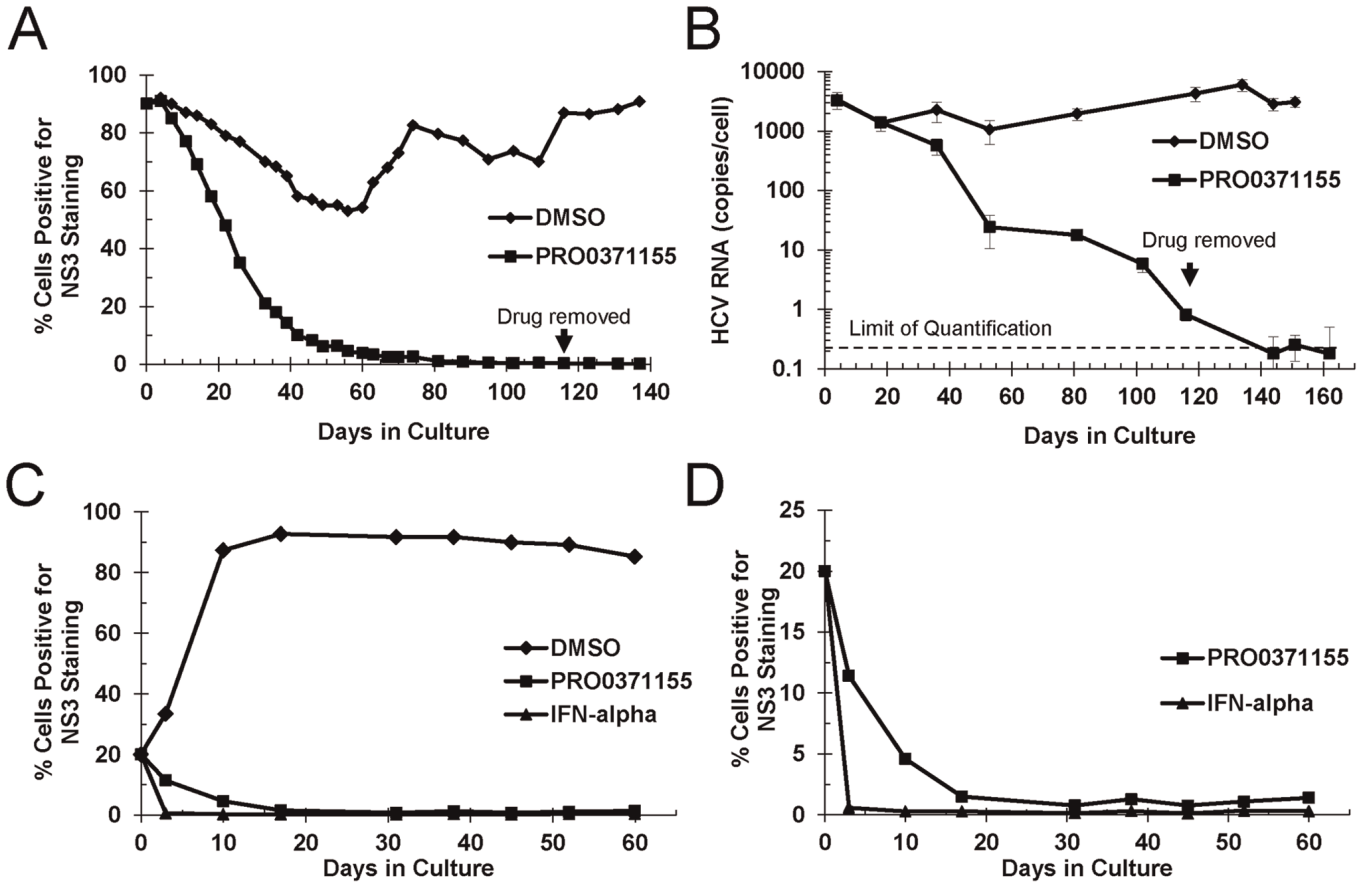
Long term treatment of HCV infected cell cultures with a triazine compound promotes viral clearance. PRO0371155 was evaluated for its inhibitory activity against GT 1a/2a HCV over multiple weeks in culture. (A) Cell cultures were ∼90% infected with cell culture-derived GT 1a/2a HCV. Cultures were treated with 1 µM PRO0371155 or DMSO in the passage control. At 3–4 day intervals, cells were subjected to staining with anti-NS3 antibodies and analysis by flow cytometry as described in the Materials and Methods. The percentage of HCV+ cells as a function of time was plotted for each experiment. (B) Results obtained in the protein expression analysis were confirmed and extended by qRT-PCR analysis of HCV RNA isolated from HCV infected cultures treated with PRO0371155 or DMSO control at various time points. HCV RNA was normalized to GAPDH mRNA and expressed in copies/cell. The limit of quantification of the qRT-PCR assay was found to be ∼0.5 copies/cell. (C) Cell cultures were ∼20% infected with cell culture-derived GT 1a/2a HCV. Cultures were treated with 1 µM PRO0371155, 10 IU/mL IFN-alpha (Sigma) or DMSO in the passage control. At 3–4 day intervals, cells were subjected to staining with anti-NS3 antibodies and analysis by flow cytometry as described in the Materials and Methods. The percentage of HCV+ cells as a function of time was plotted for each experiment. (D) Data shown in panel C were re-plotted on a different scale to accentuate the differences between IFN-alpha and PRO0371155 treated HCV+ cultures.

DNA sequences encompassing amino acid residues 171–758 of the HCV polyprotein from genotypes 3, 4, and 6 were synthesized at Bio Basic Inc. (Markham, Canada) and cloned into the *Hind*III and *Xba*I sites of the pcDNA3.1 (Invitrogen) expression vector under the control of a CMV promoter. DNA sequences were previously described and are as follows: Genotype 3, UKN3A1.9 (AY734985), Genotype 4, UKN4.11.1 (AY734986), and Genotype 6 UKN6.5.340 (AY736194) [33].

### HCV cell culture

Establishment of replicating chimeric HCV genotype 1/2a cell cultures was as described elsewhere: GT 1a/2a HCV [34] and GT 1b/2a HCV [35]. Templates for *in vitro* transcription of HCV reporter virus RNAs were designed and synthesized. Large scale RNA synthesis was performed with linearized plasmids using the RiboMAX Large Scale RNA Production System as described in the manufacturer's instructions (Promega). After RNA synthesis and removal of the DNA template, viral RNA was purified using the RNeasy Midi kit as per the manufacturer's instructions (Qiagen).

To establish a replicating HCV culture, sub-confluent human hepatoma-derived target cells obtained from Dr. F. Chisari (TSRI) [36], were resuspended in ice-cold PBS (6×10^6^ cells), mixed with *in vitro* transcribed HCV RNA and subjected to electroporation as previously described [34], [35]. Cell cultures were maintained at sub-confluent levels by passaging the cells every 3–4 days. At various days post-transfection, HCV particles from culture supernatants were harvested and clarified by centrifugation. Infection of naïve hepatoma target cells by cell culture-derived HCV was determined using the *Renilla* luciferase assay system according to the manufacturer's instructions (Promega).

### HCV viral spread assay

An assay capable of measuring both cell-free and cell-cell transmission of HCV was established based on previously published methods [37]. To evaluate cell-cell and cell-free spread of GT 1a/2a HCV, infected cells (90% HCV+) were stained with CMFDA green, according to the manufacturer's instructions (Invitrogen), and mixed at a ratio of 5∶1 with non-stained naïve cells. Mixed infected and naïve cells (7.5×10^5^) were seeded in T-75 flasks and subjected to treatment with 1 µM of a representative entry inhibitor or DMSO vehicle for 72 h at 37°C. As a positive control for cell-cell transmission, PA-25, a mouse monoclonal antibody raised against sE2 (sE2 was obtained from Austral Biologicals) was also tested in the viral spread assay. PA-25 was used at a concentration of 10 µg/mL, approximately 20-fold above its EC_50_ concentration against GT1a/2a HCV (EC_50_ = 0.5 µg/mL). Cells were detached from tissue culture flasks, washed 2× with PBS, fixed and permeabilized with BD Cytofix/cytoperm (BD Biosciences). Infected cells were stained with 10 µg/mL anti-NS3 antibody 1847 (Virostat) or mIgG1 (BD Biosciences) and counter-stained with 1 µg/mL AlexFluor 647 (Invitrogen). Single- and dual-labeled cells were quantified by flow cytometry in a FACSCalibur (BD Biosciences) and the data were analyzed using FlowJo v. 8.8.7 software (TreeStar). Long term treatment and drug resistance studies were conducted essentially as described above. Infected cell cultures (10%, 20%, 33%, 90% HCV+) were established and split every 3 days to maintain sub-confluent levels. The change in HCV+ cells, in the presence of test inhibitors or the vehicle control (DMSO), was determined every 3 days by flow cytometry as described above.

### Quantification of HCV RNA

Quantitative real-time RT-PCR assays were used to determine the abundance of viral RNA in transfected cells. Total RNA was isolated from cell lysates using the RNeasy kit (Qiagen) in accordance with the manufacturer's instructions. RNA was isolated from cell culture supernatants using the QIAamp Viral RNA kit (Qiagen). Quantitative TaqMan RT-PCR analysis was carried out using primer pairs and a probe targeting a conserved 59-base sequence within the 5′ nontranslated RNA segment of the genome: HCV19FP, CGACACTCCGCCATGAATC; HCV19RP, GCGCTTTCTGCGTGAAGAC; and HCV16 NED/MGB, NED-CCCCTGTGAGGAACTA-MGB. TaqMan assays used reagents provided with the EZ RT-PCR Core Reagent kit (Applied Biosystems) and an ABI Prism 7700 instrument. Reactions (20 µL) were incubated at 50°C for 2 min, 60°C for 45 min, and 95°C for 5 min, followed by 40 cycles of 95°C for 20 sec and 60°C for 1 min.

## Results

### Discovery of novel inhibitors of HCV entry

We optimized and validated an HCV entry assay for high throughput screening (HTS) and completed a hit finding campaign against a library of >370,000 proprietary drug-like compounds. Our screening strategy yielded multiple potent and selective hit compounds representing different chemotypes. A chemical optimization program led to the independent discovery of a series of novel inhibitors of HCV entry, represented by the generic 1,3,5-triazine structure depicted in Figure 1A [31]. A similar series of triazine inhibitors of HCV entry was recently discovered by Baldick and co-workers and published elsewhere [32].

To investigate the anti-viral properties of this series of triazines, various concentrations of representative compounds were evaluated for their ability to block entry of HCVpp into Hep3B cells. The dose response curve shown in Figure 1B represents a composite of 3 independent assays. PRO0371155 (structure shown in Figure 1A) exhibited potent activity against a prototypical genotype 1a strain in the HCVpp assay with an EC_50_ = 2 nM. In contrast, PRO0371155 did not inhibit the entry of pseudoviral particles complemented with the envelope glycoproteins from irrelevant viruses including VSV (shown in Table 1). Moreover, PRO0371155 demonstrated minimal cytotoxicity against a panel of cell lines and primary cells (Hep3B; Table 1, Huh-7, 293T, HeLa, Daudi, Ramos, Jurkat, primary hepatocyte; data not shown) in the CellTiter Glo assay, indicating the potential for a large therapeutic index. Taken together, these results suggest that PRO0371155 blocks a key step in the HCV entry process (e.g. virus-host interaction or viral fusion) rather than a non-specific host function.

The breadth of anti-viral activity of PRO0371155 was assessed in the HCVpp assay against a novel panel of fusogenic envelopes representing different genotypes. The panel consisted of envelopes from 40 unique genotype 1 strains as well as 7 strains representing genotypes 2a, 2b, 3, 4 and 6. PRO0371155 exhibited potent and specific inhibition of all twenty-one genotype 1a strains tested with a median EC_50_ = 9 nM (Figure 2A and Table 1). In contrast, PRO0371155 exhibited limited activity against genotype 1b (median EC_50_>825 nM; n = 12 strains) and genotype 4 strains (EC_50_ = 190 nM; n = 1 strain) (Figure 2A and Table 1) and no activity against HCVpp typed with envelopes representing genotypes 2a, 2b, 3, and 6 (EC_50_>1000 nM). Further chemical modification of the triazine series led to a sub-series of inhibitors that possess broad and potent activity against genotype 1a and 1b HCV strains, summarized in Table 1. A comparison of the genotype spectrum of PRO0371155 with PRO0502797 (Figure 2A and 2B) demonstrates a significant shift in terms of breadth of activity and potency across multiple genotype 1b strains in this sub-series. PRO0502797 maintained excellent activity against genotype 1a strains (median EC_50_ = 13 nM; n = 19 strains) while exhibiting potent activity against genotype 1b (median EC_50_ = 5 nM; n = 17 strains) (summarized in Table 1). PRO0502797 exhibited no activity against genotypes 2–6 in the HCVpp assay (Figure 2B). This high degree of specificity for HCV genotype 1 was also observed for a similar series of triazines [32]

The anti-viral activity and genotype specificity of the lead compounds were confirmed and extended in an authentic HCV replication assay [34]. Viral supernatants were harvested from producer cultures and used to infect naïve target cells in the presence of various concentrations of entry inhibitors. As observed in the HCVpp assay, PRO0371155 demonstrated potent anti-viral activity against GT 1a/2a HCV in culture with an EC_50_ = 9 nM (shown in Figure 1B) and no anti-viral activity against GT 1b/2a HCV (summarized in Table 1) at the highest concentration tested. A sub-series of lead compounds, exemplified by PRO0502797, were equipotent inhibitors of both HCVpp and cell culture derived HCV representing both genotypes 1a and 1b (summarized in Table 1).

Recent studies have suggested that HCV can spread in culture by both cell-free virus (*de novo*) and cell-to-cell transmission [37], [38]. To determine whether the triazine series can block both routes of infection, we utilized a flow cytometry-based cell-to-cell transmission assay [37]. Infected cells, stained with CellTracker green, were mixed with naïve cells in the presence or absence of entry inhibitors. After staining with anti-NS3 specific mAbs, newly infected cells appear as a population of singly stained cells (red) localized to the lower right quadrant in each panel shown in Figure 3. Treatment of cultures with neutralizing concentrations of the anti-E2 monoclonal antibody, PA-25, reduced but did not eliminate viral transmission (Figure 3, compare 18.3% to 5.8% HCV+ cells in the PA-25 and DMSO control, respectively). In the presence of PRO0371155, however, viral spread was reduced to background levels, demonstrating a complete block in both cell-free and cell-cell viral transmission (Figure 3, compare HCV+ cells in the lower right quadrants between PRO0371155 (0.48%) and Background (0.31%)).

### Long term treatment *in vitro* with a representative triazine compound

Small-molecule inhibitors that target the HCV NS3 protease or NS5B polymerase have been shown to clear HCV replicons from permissive cell lines [39], [40]. To test whether an HCV entry inhibitor could promote viral clearance *in vitro*, we developed a long term viral replication assay using cell culture derived GT 1a/2a HCV. The percentage of infected hepatocytes in HCV+ patients is estimated to be around 5–20% [41]–[43]. We established several HCV cell cultures that approximated this estimated value: 10%, 20%, 33% and 90%, as determined by NS3 staining. Infected cultures were passaged in the presence of PRO0371155 (∼60× EC_50_) or vehicle control, stained with anti-NS3 antibodies and analyzed by flow cytometry. In the most stringent example, infected cells treated with PRO0371155 were eradicated from a culture that was initially 90% HCV+, but not in the passage control treated with DMSO (Figure 4A). At day 116, the drug pressure was released and the cultures were passaged for an additional 30 days in the absence of PRO0371155 (see arrow in Figure 4A). Viral rebound was not observed during the washout period, indicating that the cell cultures had been completely cleared of replicating HCV (Figure 4A).

Results obtained in the flow cytometry based assay were confirmed and extended by quantitative analysis of HCV RNA (Figure 4B). After 100 days in the presence of PRO0371155 in cell cultures, HCV RNA levels were reduced to the limit of quantification in our assay (<0.5 copies/cell), as determined by serial dilution. Following removal of PRO0371155, HCV RNA was maintained at background levels for >50 days in culture, confirming the results obtained in the flow cytometry-based NS3 expression studies (Figure 4A). Our results suggest that maintenance of an infected cell culture requires multiple rounds of infection to counterbalance the loss of infected cells due to virus-induced cell death [44], [45]. Moreover, these results imply that entry inhibitors have the potential to contribute to the eradication of viral infection *in vivo*.

A second HCV cell culture was established where 20% of the cells were HCV+ at baseline. As expected, exposure of this culture to IFN-alpha resulted in a rapid and pronounced loss of NS3 staining (Figure 4C). In contrast, PRO0371155 treatment resulted in a more gradual decline in the percentage of HCV+ cells (Figure 4C), while viral spread rapidly occurred in the vehicle-treated control culture. The data for IFN-alpha and PRO0371155, re-plotted using a different y-axis scale to accentuate the different kinetics, is shown in Figure 4D. The half-life of the infected cells ranged from between 5 days (20% HCV+ culture) to 20 days (90% HCV+ culture) which is in range with the 1.7–70 days initially estimated by modeling of HCV viral dynamics *in vivo* [46].

### Drug resistance studies with PRO0371155

**Figure 5:**
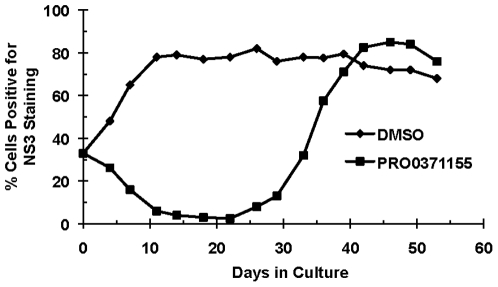
Selection of viral variants with reduced susceptibility to triazines. Cell cultures that were ∼30% infected with cell culture-derived GT 1a/2a HCV were established and treated with 1 µM PRO0371155 or DMSO in the passage control. Cultures were split twice weekly to maintain sub-confluent levels. The percentage of HCV+ cells as a function of time was determined at 3–4 day intervals. Cells were subjected to staining with anti-NS3 antibodies and analysis by flow cytometry as described in the Materials and Methods.

In our long term treatment studies, only one of four drug-treated cultures developed resistance to PRO0371155. In this culture, 33% of the cells were initially HCV positive by NS3 staining (Figure 5, day 0). In the passage control culture (DMSO), HCV rapidly spread throughout the culture to infect >90% of the total cells (Figure 5, day 14). In contrast, the percentage of infected cells in the PRO0371155-treated culture dropped rapidly, to near background levels, over 14 days of drug exposure. At day 21, viral breakthrough was observed in the presence of PRO0371155 (Figure 5) and eventually reached levels that were comparable to the untreated passage control culture (Figure 5). In this culture, loss of viral suppression and the emergence of drug resistance required ∼3 weeks, indicating a relatively moderate barrier to resistance *in vitro* (Figure 5).

Viral sequences encoding the E1/E2 envelope glycoproteins were isolated from day 42 cell cultures, cloned and sequenced. A single amino acid change, V719G, localized to the N-terminal region of the transmembrane domain of the E2 glycoprotein was found to be sufficient to confer resistance to PRO0371155 in the HCVpp assay (Table 2). Substitution of V719 with a glycine residue resulted in a >500-fold loss in susceptibility to PRO0371155 in the HCVpp assay (Table 2, compare EC_50_ = 2 nM vs. EC_50_>1,000 nM). The V719G substitution had a similar >200-fold reduction on the susceptibility of HCVpp to PRO0502797, a more broadly active triazine inhibitor (Table 2). We have also conducted a drug resistance study with PRO0502797 and have identified the identical V719G substitution as the major determinant of resistance. Genetic drift present in the culture prior to compound addition did not contribute to PRO0371155 resistance (Table 2, compare parent GT1a envelope with that of the GT1a passage control). A second amino acid change, T388A, was identified in the drug treated cultures. This variant, which localized to the E1 glycoprotein, did not have any influence on susceptibility to PRO0371155 in the HCVpp assay (Table 2). Our findings, using novel triazine inhibitors, provide an independent confirmation that V719G is the major determinant of resistance to the triazine series of HCV entry inhibitors [32].

**Table 2:** V719G is sufficient to confer resistance to representative triazines in the HCVpp assay.

Cmpd. ID	GT1a (EC_50_)	GT1a Pass. Ctrl. (EC_50_)	T388A (EC_50_)	V719G (EC_50_)
JS-81	41 ng/mL	34 ng/mL	67 ng/mL	33 ng/mL
PRO0371155	2 nM	4 nM	2 nM	>1,000 nM
PRO0502797	5 nM	n/d	n/d	>1,000 nM

### Fusogenic properties and growth kinetics of natural and drug-induced polymorphisms

**Figure 6:**
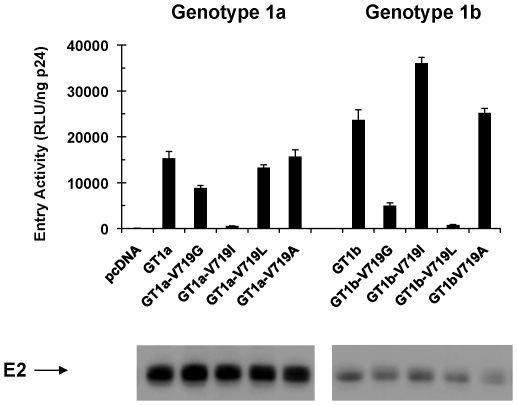
Fusogenic properties of variant envelopes and sensitivity to representative triazines in the HCVpp assay. Individual variants, representing both drug-selected and natural polymorphisms, were introduced by site directed mutagenesis of plasmid vectors that express either the genotype 1a or 1b E1/E2 glycoproteins. Unique HCVpp, representing each variant were produced in 293T cells and normalized by p24 ELISA assay. Viral entry was measured by quantifying luciferase activity as described in the Materials and Methods. Luciferase activity was expressed as R.L.U. per nanogram of p24. Cell lysates were prepared from 293T cells that were previously co-transfected with envelope expression constructs and the HIV-1 vector. After normalizing for total protein content, proteins were then separated by electrophoresis through an SDS-polyacrylamide gel and subjected to western blot analysis with PA-25, a mouse monoclonal antibody raised against sE2 derived from a genotype 1a strain.

An analysis of non-redundant HCV envelope sequences in the public database revealed that V719 is relatively well conserved (∼85%) across all genotypes [32]. Three natural polymorphisms, however, were identified at position 719, including: isoleucine (14%), leucine (<1%) and alanine (<1%) [32]. To evaluate the fusogenic properties of the potential variant envelopes, individual amino acid variants (V719G, V719I, V719L, and V719A) were introduced into a genotype 1a or genotype 1b background by site-directed mutagenesis. HCVpp, representing each potential polymorphism at position 719, were produced in 293T cells as described in the Materials and Methods, and entry activity was quantified in Hep3B cells. Luciferase activity was normalized to p24 content in the viral supernatants and expressed as R.L.U./ng p24 (Figure 6).

Introduction of the V719L amino acid variant into a genotype 1a background had no effect on HCVpp entry, while the V719G drug-induced variant exhibited a modest 1.5-fold reduction in entry activity compared to the parental GT 1a strain (Figure 6, compare GT 1a with GT 1a-V719L and GT 1a-V719G). In contrast to these results, GT 1a-V719I exhibited a 30-fold reduction in HCV entry activity (Figure 6, compare GT 1a with GT 1a-V719I). Surprisingly, the opposite pattern was observed after identical substitutions were introduced into the GT 1b background. In this case, introduction of V719G and V719L resulted in a 5- and 30-fold reduction in HCV entry activity compared to the parental GT 1b strain, respectively (Figure 6, compare GT 1b with GT 1b-V719G and GT 1b-V719L), whereas the V719I substitution resulted in a modest 1.5-fold enhancement of entry (Figure 6, compare GT 1b with GT 1b-V719I). In agreement with these findings, I719 represents a significant fraction (21%; total of n = 438 non-redundant sequences) of genotype 1b sequences in the public database and is identified in only a small fraction of genotype 1a sequences (0.9%; total of n = 797 non-redundant sequences) [47].

Other polymorphisms were found to be extremely rare: L719 (GT 1a: 0.1%; n = 797 and GT 1b: 0%; n = 438) and both G719 and A719 were not found in our survey of 1,235 genotype 1 sequences in the database [47]. The entry properties of each of the potential polymorphisms at position 719 partly explain the frequency of occurrence in the natural HCV genotype 1 population (Figure 6). However, the relatively high levels of HCVpp entry activity observed for the V719G, V719L, V719A variants in the GT 1a background and V719A in the GT 1b background (see Figure 6) did not correlate with their low frequency of occurrence in the natural population. Our results suggest that properties, other than an effect on viral entry, (e.g. virus assembly and release) may be responsible for the reduced fitness and low frequency of these specific amino acid polymorphisms in the natural HCV genotype 1 population [47].

To further evaluate the relative fitness of these polymorphisms, we successfully introduced each amino acid variant into the full-length HCV GT 1b/2a reporter construct by site-directed mutagenesis. We also successfully introduced V719G and V719A into the GT 1a/2a HCV background, but were unable to generate stable clones representing V719I and V719L in this background. Full-length reporter RNA transcripts were prepared, purified and electroporated into target cells. Viral supernatants were sampled every 3–4 days from long term HCV cell cultures. Growth kinetics were determined by quantification of *Renilla* luciferase activity following a 72 hr infection of naïve target cells with supernatants harvested from long-term HCV cell cultures.

**Figure 7:**
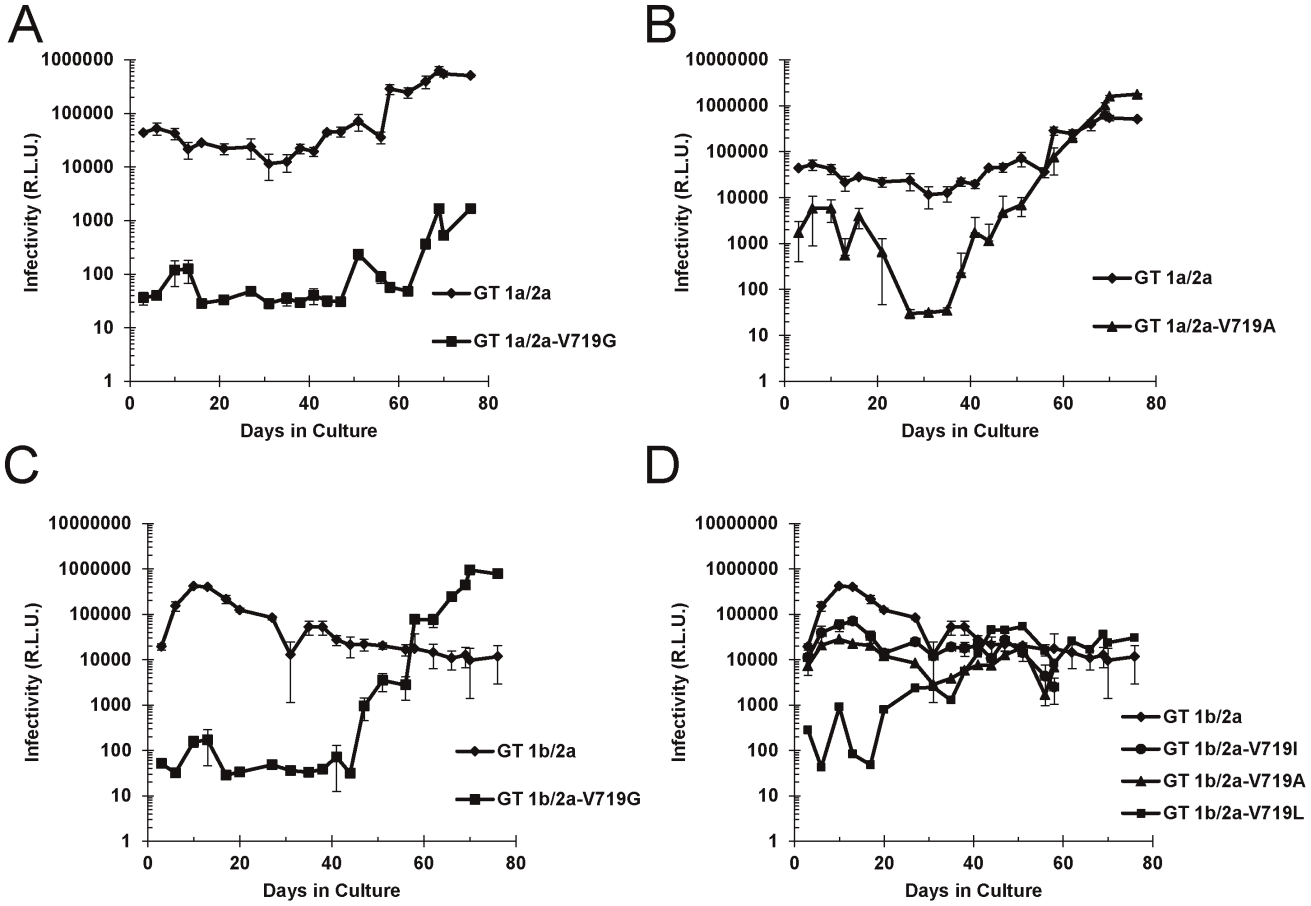
Effect of drug-induced and natural amino acid variants at position 719 on the growth kinetics of HCV genotype 1/2a in cell culture. The drug resistant variant, V719G and three natural amino acid polymorphisms, I719, L719, A719, were introduced into genotype 1a and 1b genetic backgrounds using the QuikChange II XL site-directed mutagenesis kit (Stratagene). Full-length HCV RNAs bearing various polymorphisms at position 719 were prepared and electroporated into naïve target cells. Infected cell cultures were maintained for up to 2.5 months. Every 3–4 days, viral supernatants were harvested and subsequently used to infect naïve target cells. After 72 hrs, *Renilla* luciferase activity was measured and viral titer/infectivity was expressed in R.L.U. (relative light units). Changes in viral titers were plotted on a logarithmic scale as a function of time. (A) A comparison of the growth kinetics of GT 1a/2a HCV (diamonds) with the drug-induced variant, GT 1a/2a-V719G (squares) (B) A comparison of the growth kinetics of GT 1a/2a HCV (diamonds) with a potential polymorphism, GT 1a/2a-V719A (triangles). (C) A comparison of the growth kinetics of GT 1b/2a HCV (diamonds) with the drug-induced polymorphism, GT 1b/2a-V719G (squares). (D) A comparison of the growth kinetics of GT 1b/2a HCV (diamonds) with a natural polymorphism, GT 1b/2a-V719I (circles) and two potential polymorphisms GT 1b/2a-V719L (squares) and GT 1b/2a-V719A (triangles).

Following electroporation of naïve target cells, GT 1a/2a HCV cell cultures displayed high levels of infectivity which gradually increased over time out to 76 days in culture (Figure 7A). GT 1b/2a HCV experienced a more rapid increase in *Renilla* luciferase activity reaching peak levels at day 10 and then tapering off over the following 66 days in culture (Figure 7C). The bell shaped kinetics of the GT 1b/2a HCV infection was consistent with those observed previously by others and published elsewhere [35]. Introduction of all natural and drug-induced polymorphisms into either of the GT 1a/2a or GT 1b/2a HCV backgrounds resulted in a marked reduction in viral infectivity as measured by *Renilla* luciferase gene expression at day 10. The effect on viral infectivity in culture ranged from a modest 6-fold inhibition, observed for GT 1b/2a-V719I (Figure 7D), representing the most common polymorphism, to a >2 log reduction in viral infectivity observed for both the GT 1a/2a-V719G (Figure 7A) and GT 1b/2a-V719G HCV cell cultures (Figure 7C). Taken together, the kinetics of HCV infection in culture suggests that amino acid substitutions at position 719 result in a significant decrease in viral fitness. Further experiments, however, are required to fully understand the mechanism and fitness costs of these amino substitutions at position 719.

Several cultures exhibited low level infectivity for several weeks in culture and then experienced a burst in *Renilla* luciferase activity: GT 1a/2a-V719A (at day 37) (Figure 7B), and GT 1b/2a-V719G (at day 43) (Figure 7C). Sequencing of the envelope glycoproteins from these cultures revealed the presence of additional amino acid substitutions within the E1 and E2 glycoproteins. All other viruses exhibited no change across the E1/E2 envelope sequence, with the exception of GT 1b/2a-V719I which had accumulated two additional substitutions within the envelope sequence at day 10 (see Table S1). Further studies are needed to determine whether these amino acid variants represent adaptive or compensatory mutations or are simply the result of genetic drift in the long-term HCV cell cultures.

### Drug resistance properties of variant envelopes bearing natural polymorphisms

The susceptibility of each potential variant envelope to PRO0371155 was determined in the HCVpp assay. Compared to the parent GT1a envelope, PRO0371155 was 500-fold less active against the V719L variant in the HCVpp assay (Table 3, compare EC_50_ = 2 nM (GT1a) to EC_50_ >1,000 nM GT1a-V719L). This level of resistance was similar to that observed for the V719G variant which was selected under drug pressure. Introduction of either V719I or V719A into the GT 1a envelope resulted in a more modest reduction in the potency of PRO0371155 of 34- and 46-fold respectively (Table 3).

Isoleucine is the most common natural polymorphism and represents a significant fraction of the genotype 1b sequences in the public database (21%). Interestingly, the V719I substitution had no effect on sensitivity to PRO0502797 in either the genotype 1a or 1b backgrounds (Table 3). In contrast, V719L exhibited a significant reduction in sensitivity to PRO0502797 when introduced into the genotype 1a background (Table 3) and a more modest reduction in sensitivity to PRO0502797 in the genotype 1b background (Table 3). Likewise, introduction of V719A into either of the genotype 1 backgrounds resulted in a modest reduction in sensitivity to PRO0502797 (Table 3). The overall frequency of pre-existing drug resistant polymorphisms in the HCV genotype 1 population is very low. Only I719 represents a significant fraction of sequences in the genotype 1b background and this variant is fully susceptible to inhibition by PRO0502797.

## Discussion

We have completed a hit finding campaign against a random library of diversified drug-like compounds and identified a series of 1,3,5-triazines, similar to that previously reported by Baldick and co-workers [31]. In the Baldick study, a genotype 1b specific primary screen led to the identification of hit compounds that were highly specific for HCV genotype 1b strains. In a complementary approach, we utilized a genotype 1a-based screen which led to the discovery of compounds that were exclusively active against HCV genotype 1a. Independent chemical optimization of these two series produced triazine compounds that are broadly active against both genotype 1a and 1b HCV strains. Further optimization efforts were unable to uncover inhibitors that were broadly active against other genotypes. Our studies provide an important independent confirmation of the major anti-viral properties and resistance patterns of the triazine series. We have extended previous findings through the analysis of the relative fitness of natural and drug-induced amino acid variants at position 719. We show that the most common natural polymorphism, I719, remains fully susceptible to inhibition by triazine inhibitors and that the frequency of pre-existing, naturally resistant HCV variants is extremely low in the genotype 1 background. We also demonstrate, for the first time, that a highly active entry inhibitor can lead to the eradication of an active HCV infection in cell culture.

HCV replication inhibitors have shown the ability to completely suppress viral replication, leading to viral clearance in multiple experimental systems including replicon cell lines [40], [48]. Unlike replication inhibitors, however, a rapid drop in HCV RNA and protein expression was not expected in our studies since infected cells should continue to produce viral particles in the presence of an entry inhibitor. As expected, a slower rate of HCV+ cell decline was observed in the PRO0371155 treated HCV cell cultures compared with the INF-alpha treated control. However, it was surprising to observe the complete clearance of HCV from infected cell cultures over a relatively short duration in the presence of an entry inhibitor alone. Although the mechanisms that drive turnover of infected hepatocytes are not fully understood, induction of apoptotic pathways has recently been observed in a chimeric mouse model of HCV infection [45], [49] and later confirmed in the HCV cell culture system with human hepatoma-derived cell lines [44]. The dynamics of HCV infection in culture suggest a model in which infected cells continually turnover due to apoptotic mechanisms. Consistent with this model, HCV cell cultures treated with PRO0371155 grew more efficiently and exhibited less cell death compared to untreated control cultures (data not shown). Our results suggest that multiple rounds of infection of naïve hepatocytes are likely required to maintain HCV infection in culture and, presumably, *in vivo*.

In a normal healthy liver, the rates of hepatocyte proliferation and cell death are negligible. As a result, hepatocytes are long lived and exhibit minimal turnover *in vivo* (T_1/2_∼70 days). In patients chronically infected with HCV, however, the proportion of proliferating hepatocytes increases dramatically by 100- to 1,400-fold compared to healthy controls, as determined by staining of biopsies for cell division markers [50]. Fitting patient data to mathematical models that take into account hepatocyte proliferation also suggests that during anti-viral therapy, productively infected cells are preferentially replaced by proliferating naïve, non-infected hepatocytes [43]. Although our *in vitro* system is incomplete, the observed viral dynamics in cell culture provides some experimental support for this hypothesis. In the presence of inhibitory concentrations of PRO0371155, the rate of infection approaches zero. Under these conditions, non-infected naïve cells proliferate and eventually dominate the cell culture population. The implications of these results are two-fold: 1) HCV infected cells die at a faster rate than non-infected cells in culture and 2) HCV infected cells proliferate at a slower rate than non-infected cells in culture. A contrasting picture is observed in control cultures that lack PRO0371155. In this case, the rate of infection of naïve target cells is greater than the rate of cell death of productively infected cells. As a result, HCV spreads rapidly to infect nearly 90% of the cells in culture. Moreover, these cell cultures remain stably infected over multiple months. Our results demonstrate that long term exposure to a potent entry inhibitor can lead to the eradication of HCV *in vitro*.

**Table 3:** Natural polymorphisms at position 719 confer resistance to representative triazines in the HCVpp assay.

Background	Envelope	JS-81 (EC_50_)	PRO0371155 (EC_50_)	PRO0502797 (EC_50_)
Genotype 1a	GT 1a	48 ng/mL	2 nM	5 nM
	V719L	97 ng/mL	>1,000 nM (500×)	625 nM (125×)
	V719I	62 ng/mL	67 nM (34×)	5 nM (1×)
	V719A	53 ng/mL	92 nM (46×)	35 nM (7×)
Genotype 1b	GT 1b	50 ng/mL	317 nM	3 nM
	V719L	41 ng/mL	>1,000 nM (3×)	27 nM (9×)
	V719I	78 ng/mL	>1,000 nM (3×)	3 nM (1×)
	V719A	30 ng/mL	273 nM (0.9×)	12 nM (4×)

To date, only one oral HCV entry inhibitor with a defined mechanism of action, ITX-5061 (iTherX), has entered clinical testing (Phase 2a) [51]. ITX-5061 binds directly to SR-B1, a known HCV co-receptor, and blocks a key post-binding step in the viral entry process [51]. Preliminary mechanism of action studies indicate that the triazines also target the HCV envelope at a post-attachment step in the entry process (data not shown). However, triazines do not block binding of soluble E2 to cell lines expressing CD81 and/or SR-B1 in neutralization of binding experiments (data not shown). The triazine series, therefore, exhibits a distinct mechanism of action compared to ITX-5061 and is likely to possess a complementary pattern of resistance. Our drug resistance studies directly implicate the transmembrane domain of E2 as a potential target for the triazine series. The partially buried location of V719 near the surface of the viral membrane fuels speculation that the triazines may interfere with HCV fusion. Further studies are required to test this hypothesis and to fully define the molecular target and mechanism of action of this inhibitor series.

Previous models of HCV viral dynamics have provided important information into ribavirin's mechanism of action and contribution to combination therapy with pegylated interferon-alpha [52]. These models may also provide important insights into the potential role of an entry inhibitor in a future combination therapy for HCV infection. Therapeutic intervention with pegylated interferon-alpha typically results in a biphasic pattern of HCV RNA decay [46]. The first phase of viral decay represents the rapid clearance of HCV particles from the plasma compartment following inhibition of replication. The second, slower phase is governed by the rate of infected hepatocyte cell death, counterbalanced by the rate of *de novo* infection and the rate of proliferation of productively infected cells [46], [53]. Ribavirin has been shown to accelerate the second phase of viral decay in a dose-dependent manner when the response to pegylated interferon-alpha is low [52]. Although mechanistically distinct from ribavirin, entry inhibitors would be expected to similarly affect the second phase of HCV decay by virtue of blocking new rounds of HCV infection [43], [52]. Models of HCV viral dynamics support the addition of an entry inhibitor to HCV combination therapy to improve SVR rates and prevent the establishment and outgrowth of drug-resistant viruses in a manner similar to that observed for ribavirin.

New anti-viral drugs, with complementary mechanisms of action, are needed to reliably cure patients of HCV infection and to reduce the toxicity and duration of treatment. Viral entry is a validated target for other highly mutable viruses and represents a novel and attractive approach to advance HCV therapy. We have discovered and optimized a series of triazine compounds that are potent, selective and non-cytotoxic inhibitors of HCV entry. Compounds from this series have demonstrated high anti-viral activity against HCV *in vitro*, including the ability to eradicate HCV from infected cell cultures. These findings support further investigation of entry inhibitors as a potential new class for HCV therapy.

## Supporting Information

Table S1 HCV cell cultures representing various drug-induced and natural amino acid polymorphisms were established as described in the legend to Figure 7. Once peak viral titers were achieved, HCV RNA was isolated from each culture and the E1/E2 glycoprotein coding sequence was amplified by RT-PCR and subjected to direct DNA sequencing. Amino acid substitutions identified in the E1 or E2 glycoproteins are summarized in Table S1. The time to peak titers and associated sequencing data are also denoted in Table S1. Following electroporation of naïve target cells, GT 1a/2a-V719A HCV initially exhibited 1–2 log lower Renilla luciferase activity compared to the parent GT 1a/2a HCV background. At day 40, however, the GT 1a-V719A HCV cell culture experienced a burst in luciferase activity. Sequencing of the E1/E2 envelope glycoproteins from day 76 cultures revealed the presence of one additional amino acid variant, D263E which is localized to the N-terminal region of a hydrophobic domain in E1 (aa262–290) that houses a putative fusion peptide sequence. No changes were observed in the E1/E2 envelope sequences from either the parent GT 1a/2a HCV or the GT 1a/2a-V719G HCV variant cultures as determined by sequencing of day 69 and 76 cultures, respectively. In contrast to the GT 1a/2a HCV cell culture, the parent GT 1b/2a HCV showed a distinct pattern of infectivity in cell culture. In this case, Renilla luciferase activity rapidly reached a maximum level at day 9–10, tapering off over the following 67 days in culture. The GT 1b/2a-V719I and GT 1b/2a-V719G HCV variants exhibited 6- and 20-fold reduced levels of Renilla luciferase activity compared to the parent GT 1b/2a HCV background, respectively, while the GT 1b/2a-V719G and GT 1b/2a-V719L HCV variants exhibited a more dramatic, 2–3 log, reduction in viral titers. Sequencing of day 10 cultures revealed the presence of two additional amino acid variations in the GT 1b/2a-V719I HCV sequence. One substitution, A357T, was localized to the transmembrane domain of E1 (aa353–381) while the second substitution, A746P, was located at the immediate C-terminus of the E2 protein near the transmembrane domain. No other changes were identified by sequencing of the GT 1b/2a-V719L (day 51), GT 1b/2a-V719A HCV (day 9) variants or the parent GT 1b/2a HCV background (day 9). The GT 1b/2a-V719G HCV variant exhibited low levels of infectivity compared to the parent GT 1b/2a HCV at day 9–10. A significant burst in Renilla luciferase activity was observed between days 43–50. Sequencing at day 69 revealed two additional amino acid substitutions including: A217E and A457G located in the E1 and E2 glycoproteins, respectively. Surprisingly, viral titers of HCV GT 1b/2a reporter constructs bearing these amino acid substitutions reached and surpassed the peak levels observed for the parental GT1 b/2a HCV strain. Additional experiments are necessary to determine whether these amino acid variants represent adaptive or compensatory mutations or are simply the result of genetic drift in the long-term HCV cell cultures. Moreover, the potential role of these amino acid positions on viral entry, gene expression, and virus assembly/release requires additional study.
(DOC)
