# Supplementary material for: Novel Small-Molecule Inhibitors of Hepatitis C Virus Entry Block Viral Spread and Promote Viral Clearance in Cell Culture
Source: PLoS One. 2012 Apr 24;7(4):e35351. doi: 10.1371/journal.pone.0035351 (PMC3335862; doi:10.1371/journal.pone.0035351)
Supplement: Table S1 — HCV cell cultures representing various drug-induced and natural amino acid polymorphisms were established as described in the legend to Figure 7. Once peak viral titers were achieved, HCV RNA was isolated from each culture and the E1/E2 glycoprotein coding sequence was amplified by RT-PCR and subjected to direct DNA sequencing. Amino acid substitutions identified in the E1 or E2 glycoproteins are summarized in Table S1. The time to peak titers and associated sequencing data are also denoted in Table S1. Following electroporation of naïve target cells, GT 1a/2a-V719A HCV initially exhibited 1–2 log lower Renilla luciferase activity compared to the parent GT 1a/2a HCV background. At day 40, however, the GT 1a-V719A HCV cell culture experienced a burst in luciferase activity. Sequencing of the E1/E2 envelope glycoproteins from day 76 cultures revealed the presence of one additional amino acid variant, D263E which is localized to the N-terminal region of a hydrophobic domain in E1 (aa262–290) that houses a putative fusion peptide sequence. No changes were observed in the E1/E2 envelope sequences from either the parent GT 1a/2a HCV or the GT 1a/2a-V719G HCV variant cultures as determined by sequencing of day 69 and 76 cultures, respectively. In contrast to the GT 1a/2a HCV cell culture, the parent GT 1b/2a HCV showed a distinct pattern of infectivity in cell culture. In this case, Renilla luciferase activity rapidly reached a maximum level at day 9–10, tapering off over the following 67 days in culture. The GT 1b/2a-V719I and GT 1b/2a-V719G HCV variants exhibited 6- and 20-fold reduced levels of Renilla luciferase activity compared to the parent GT 1b/2a HCV background, respectively, while the GT 1b/2a-V719G and GT 1b/2a-V719L HCV variants exhibited a more dramatic, 2–3 log, reduction in viral titers. Sequencing of day 10 cultures revealed the presence of two additional amino acid variations in the GT 1b/2a-V719I HCV sequence. One substitution, A357T, was localized to th [file pone.0035351.s001.doc]

**Table S1: Potential adaptive mutations identified in long term cultures of GT 1/2a HCV variants bearing drug-induced and natural polymorphisms at position 719**
